# Referral for Intensive Home Treatment or Psychiatric Inpatient Care? A Retrospective, Observational Comparison of Patient and Process Characteristics

**DOI:** 10.3389/fpsyt.2022.875495

**Published:** 2022-05-25

**Authors:** G. C. Roselie van Asperen, André I. Wierdsma, Remco F. P. de Winter, Cornelis Lambert Mulder

**Affiliations:** ^1^Parnassia Psychiatric Institute, Rotterdam, Netherlands; ^2^Epidemiological and Social Psychiatric Research Institute, Department of Psychiatry, Erasmus University Medical Center, Rotterdam, Netherlands; ^3^Department of Clinical, Neuro and Developmental Psychology, Vrije Universiteit Amsterdam, Amsterdam, Netherlands; ^4^Mental Health Institute Rivierduinen, Leiden, Netherlands

**Keywords:** intensive home treatment, emergency psychiatry, acute psychiatry, psychiatric inpatient care, referral

## Abstract

**Introduction:**

Intensive home treatment (IHT) is intended to prevent the (mostly voluntary) admission of mentally ill patients by providing intensive care in their domestic environment. It requires approaches to referral that ensure the delivery of the best possible acute care. Indications for referral may be improved by greater understanding of the clinical profiles of patients referred for IHT and of those referred for inpatient care. As such understanding may also further the development of IHT and innovations within it, we compared the patient and process characteristics associated with IHT referral for those associated with inpatient care.

**Methods:**

This retrospective, observational, explorative study was conducted from 2016 to 2019. Patients aged 18 years and older were assessed by the emergency psychiatric outreach services in the greater Rotterdam area (Netherlands). Anonymized data were used to compare patient and process characteristics between patients referred for IHT and those admitted voluntarily. Patient characteristics included gender, age, cultural background, living situation and main diagnosis. Additional the case mix was measured using the Severity of Psychiatric Illness (SPI) scale. Process characteristics included psychiatric history, the total number of contacts with the emergency psychiatric outreach services, assessments during office hours, place of assessment, referrer, and the reason for referral. Using multiple logistic regression analysis, the patient and process characteristics associated with IHT referral were compared with those associated with voluntary admission.

**Results:**

The emergency psychiatric outreach services undertook 12,470 assessments: 655 were referred for HT and 2,875 for voluntary admission. Patient characteristics: referral for IHT rather than voluntary admission was associated with higher motivation for treatment and better family involvement. Process characteristics: referral for IHT rather than voluntary admission was associated with assessment by the crisis services within office hours, no mental health treatment at the time of referral, and referral by a family doctor.

**Discussion:**

IHT in a specific Dutch setting seems to function as an intensive crisis intervention for a subgroup of patients who are motivated for treatment, have social support, and are not in outpatient treatment. The patient and process characteristics of patients referred for IHT should now be studied in more detail, especially, for having more social support, the role of the family members involved.

## Introduction

Intensive home treatment (IHT) is a Dutch offshoot of crisis resolution and home treatment (CRHT) in mental health care, an approach that originated in the United Kingdom (1, 2). Its main aim, similar to CRHT, is to prevent the hospital admission and provide facilitated discharge from inpatient wards of severe mentally ill patients by treating them intensively at home for 7 days a week, also outside office hours.

The intensive care provided in IHT consists of a range of approaches such as pharmacotherapy, therapeutic treatment, systemic treatment, helping patients to improve their insights into the perpetuation of symptoms, and providing them with guidance on problems in their direct living environment. This care is provided by a multiprofessional team in the patient's domestic environment, and is available around the clock (3).

The outcomes of IHT on preventing hospital admission were investigated in two earlier randomized controlled trials (RCTs). The first, in the United Kingdom, showed a reduction in the number of voluntary admissions (4). The second, in Switzerland, did not show a reduction in admissions, but showed a reduction in the number of hospital bed days (5). The outcomes of IHT on providing facilitated discharge were investigated in an observational study in the United Kingdom (6). The results did not show a reduction in readmissions, but did show a reduction in the number of hospital bed days.

Both RCTs indicated that it was not easy to conduct the thorough triage needed for IHT, which was still a relatively new intervention for triage staff, and that patients needed immediate treatment. The aim of triage is to determine whether patients referred for triage are “suitable” for IHT. According to local choices and local organization, triage can be done by the IHT professionals themselves, by the professionals who refer patients for IHT (such as those at emergency psychiatric outreach services), or by a combination of the two. To make it possible in all cases for the best possible acute care to be delivered, professionals need to indicate the need for IHT correctly—in other words, IHT when it is possible and inpatient care when it is needed (5).

Studies to the characteristics of patients referred for IHT showed different outcomes. An observational study of IHT patients in the Netherlands found that, at the time of referral, 51% were undergoing an exacerbation of their mental illness, and that 22% had increased suicidal intention. At 66%, affective disorder was the most common diagnosis (7). A retrospective descriptive study in Ireland found that 44% of patients referred for IHT suffered from mood disorders and 14% from suicidal intention (8). An observational study of Home Treatment vs. inpatient care in Switzerland found HT patients had more often an affective disorder and less often a substance use disorder (9).

Two earlier studies have investigated the factors associated with the transfer from IHT back to inpatient care (10, 11). Patients treated by an IHT like team were more likely to be admitted when they had previous hospital admission (10, 11), had high suicidal ideation (11), were at risk of self-neglect, had been uncooperative with initial assessment, had been assessed outside office hours and had been assessed in hospitality casualty departments (10).

The findings of an observational study to the patient and process characteristics of patients treated by a home treatment team compared to patients treated on hospital wards in Switzerland (12) found a primary diagnosis of anxiety or stress-related disorder, employment status and the degree of social problems affected the probability of whether or not HT was initiated. Almost all of the patients in this study were initially treated on hospital wards, where these results applied mostly to the aim of facilitated discharge and less to prevent hospital admission.

Appropriate referrals by trained professionals prevent discussion about acceptance and time spent on assessing patients who are not considered suitable (13). Unnecessary hospitalization can be prevented (14). For professionals who perform the triage, it is helpful to be familiar with the differences between the clinical profiles of patients referred for IHT and those of patients referred for inpatient care. The resulting understanding would help develop more fully reasoned procedures for improving the referral process. It also may help to develop IHT and improve IHT policy. The aim of this study was therefore to explore the patient and process characteristics associated with referral for IHT and inpatient care.

## Methods

### Study Design

Using a retrospective, observational, explorative study design, we explored the patient and process characteristics of referrals by the emergency psychiatric outreach services to either IHT or inpatient care. Because IHT is usually viewed as an alternative to voluntary admission (3), we compared patients referred for IHT with those referred for voluntary admission.

### Setting and Participants

**Figure 1:**
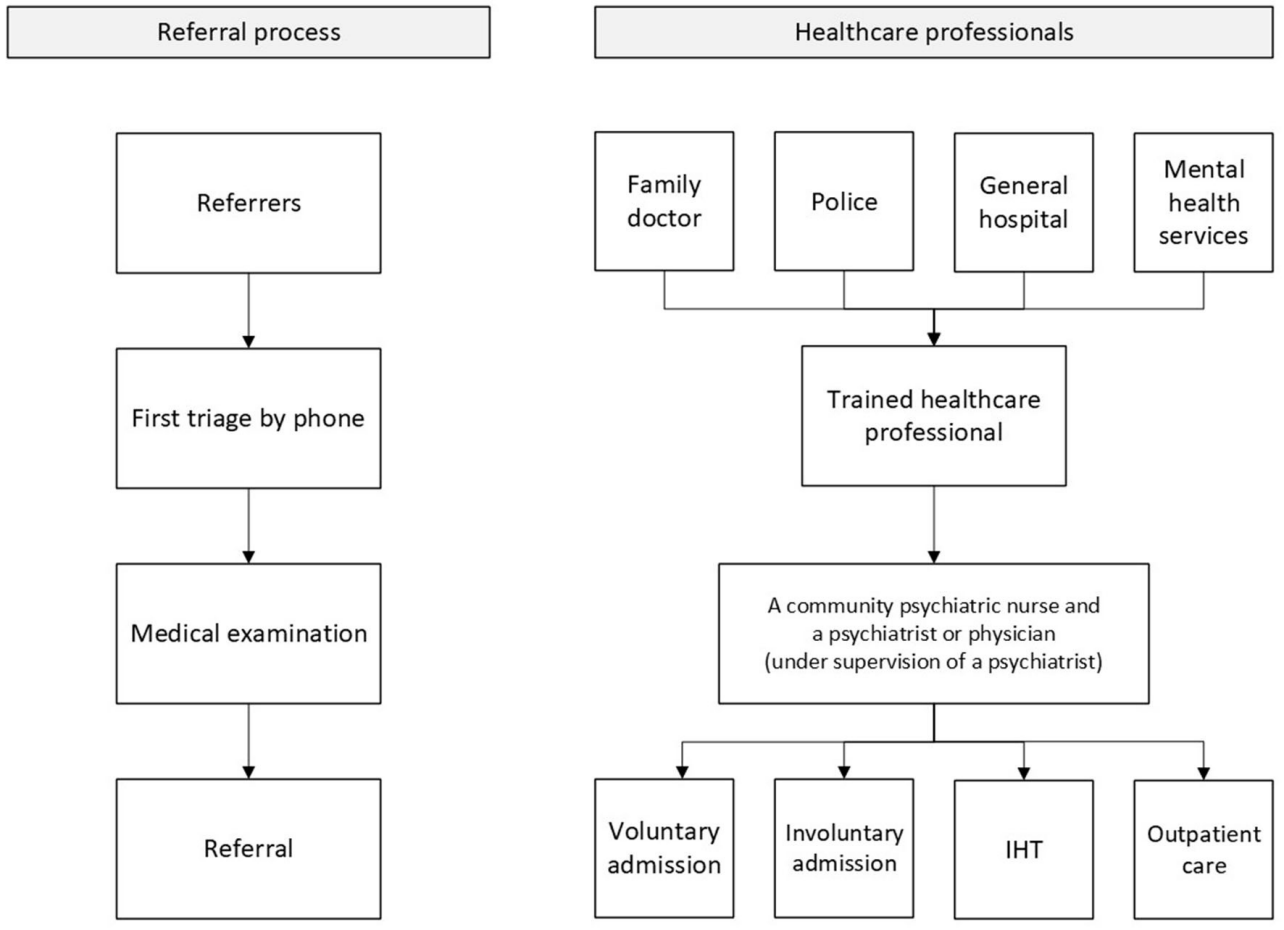
Referral process.

The study was conducted in the greater Rotterdam area (southwestern Netherlands), where IHT and emergency psychiatric outreach services are provided by separate departments working in close collaboration. Figure 1 shows the referral process. Patients are first referred—mainly by a family doctor, the police, a general hospital, or the mental health services—to the emergency psychiatric outreach services, whose primary tasks are triage, first aid and referral (15, 16). The outreach services are responsible for responding rapidly to sudden changes in patients' mental health, or to their loss of behavioral control, including suicidal crises. First triage take place by a trained healthcare professional to assess if an examination is needed. The medical examination is carried out by two mental healthcare professionals: a community psychiatric nurse and a psychiatrist or physician (under supervision of a psychiatrist), who is responsible for making a psychiatric diagnosis. However, as these services do not assess every single patient who is considered for inpatient care, Rotterdam lacks the gatekeeping role that is central to the original CRHT model (4).

The patients included in this study were aged 18 and above, they had been assessed by the emergency psychiatric outreach services in the 2016-2019 period. They were referred for (in)voluntary admission in one of the psychiatric hospitals in Rotterdam, to one of the two IHT teams in Rotterdam or back to outpatient care. IHT Rotterdam delivered care based on the Dutch IHT model (3). Both teams consisted of a psychiatrist and psychiatric nurses, who delivered care 7 days a week between 8 a.m. and 10 p.m. The emergency psychiatric outreach services were available by phone and for emergency visits during 10 p.m. and 8 a.m., Being motivated for treatment was in inclusion criteria. Being homeless and having a substance-related disorder as main diagnosis were exclusion criterions for IHT.

### Data Collection

This study was conducted in accordance with Dutch law, and particularly with the Medical Research Involving Human Subjects Acts (WMO) (17), which did not require the specific approval of the regional medical ethical committee. The reporting was in accordance with the STROBE (Strengthening the reporting of observational studies in epidemiology) Statement (18).

Data were obtained retrospective based on routine medical date from the WebRAAP (acronym in Dutch: Web Registratie- en Adviessysteem voor de Acute Psychiatrie) electronic health record (EHR), which applies specifically to acute mental health care (19). Before they were accessed by the authors, these data were fully anonymized by Myosotis, a data-processing company. Data was obtained on the following patient characteristics: gender, age, cultural background, living situation, main diagnosis, and the case mix. The Severity of Psychiatric Illness (SPI) scale was used to explore the case mix. The 14 dimensions of the SPI assess the severity of psychiatric illness and were rated on a 4-point scale from 0 to 3, with 0 indicating no problems and 3 indicating an extreme problem (20, 21). Because data were distributed asymmetrically we dichotomized the variables as “no problem” vs. “small to severe problem.” The 14 dimensions are: suicide potential, danger to others, severity of psychiatric symptoms, problems with self-care, substance abuse, medical complications, social complications, problems with professional functioning, problems with living conditions, problems with motivation for treatment, problems with compliance, problems with disease awareness, problems with family involvement, and persistence of problems. To describe the process characteristics, data collection included the following: psychiatric history, total number of contacts with the emergency psychiatric outreach services, assessment during office hours, place of assessment (home, police station, family doctor, general hospital, or psychiatric hospital), type of referrer (family doctor, police, or general hospital), and reason for referral (depressive symptoms, psychotic symptoms, danger to others, and/or danger to themselves).

### Data Analysis

Statistical analyses were performed using SPSS (Statistical Package for the Social Sciences) version 26.0 (SPSS Inc., Chicago, IL). Descriptive statistics were used to summarize patient and process characteristics. Predictors chosen to construct the analyses were based on the factors found in the literature associated with admission to psychiatric care vs. IHT. Multiple logistic regression analysis was performed to compare the associations of IHT referral and voluntary hospital admission with demographic and clinical factors. Model selection was based on Wald tests with alpha set at 5%. All models included three case-mix variables: age, gender, and severity of psychiatric symptoms. The fit of the final model was assessed with the Hosmer-Lemeshow goodness-of-fit statistic and the area under the receiver operating characteristic (ROC) curve.

## Results

During the 2016-2019 period, the emergency psychiatric outreach services undertook 12,470 assessments of patients aged 18 and older, leading 655 (5.3%) to be referred for IHT. A minority of the referrals were multiple referrals (12%): 579 were unique patients and 76 multiple referrals. From the multiple referrals, 48 patients were referred twice, 11 patients were referred three times and 2 patients were referred four times. Over this period, the total number of patients referred for IHT increased from 67 (2016) to 313 (2019). In total, 2,875 patients (23.0%) were referred for voluntary admission. A majority of the other 8,940 patients (71.7%) were referred for outpatient care or for involuntary admission, these patients were not included in our analysis

Table 1 shows the patient characteristics of the patients referred for IHT and voluntary admission. Proportionally, more women were referred for IHT. Patients in the IHT group were also younger, and a greater number were living with their family than in the voluntary admission group. A majority of IHT patients (39.2%) were diagnosed with a depressive disorder, proportionally, a smaller group of patients were referred for voluntary admission (21.5%). Very few patients diagnosed with an organic disorder were referred for IHT, a higher proportion of them were referred for voluntary admissions.

**Table 1:** Characteristics of patients assessed by the psychiatric emergency services.

**Factors**	**IHT**	**Voluntary admission**	**Total**
Total n*	655 (5.3%)	2,875 (23.0%)	12,470 (100%)
Age: mean (SD)	40.16 (14.54)	42.57 (16.43)	43.38 (18.17)
Gender: *n*, female	392 (59.8%)	1,328 (46.2%)	5,734 (46.0%)
Living situation Alone: *n* With family: *n* Institution: *n* Other/unknown: *n* Without residence: *n*	159 (24.3%) 385 (58.8%) 1 (0.2%) 110 (16.7%) 0 (0.0%)	1,035 (36%) 934 (32.5%) 125 (4.3%) 781 (27.2%) 127 (4.4%)	4,172 (33.5%) 4,364 (35.0%) 5,74 (4.6%) 3,360 (26.9%) 465 (3.7%)
Primary diagnosis Depressive disorder: *n* Bipolar disorder: *n* Anxiety disorder: *n* Post-traumatic stress syndrome: *n* Psychosocial problems: *n* Adjustment disorder: *n* Personality disorder: *n* Psychotic disorder: *n* Organic disorder: *n* Alcohol-related disorder: *n* Other substance-related disorder: *n* Other: *n* None/diagnoses deferred: *n*	257 (39.2%) 46 (7.0%) 55 (8.4%) 28 (4.3%) 5 (0.8%) 16 (2.4%) 24 (3.7%) 190 (29.0%) 2 (0.3%) 1 (0.2%) 3 (0.5%) 27 (4.1%) 1 (0.2%)	618 (21.5%) 156 (5.4%) 95 (3.3%) 65 (2.3%) 64 (2.2%) 81 (2.8%) 271 (9.4%) 886 (30.8%) 88 (3.1%) 193 (6.7%) 152 (5.3%) 196 (6.8%) 10 (0.3%)	2,017 (16.2%) 797 (6.4%) 409 (3.3%) 312 (2.5%) 333 (2.7%) 442 (3.5%) 1,112 (8.9%) 3,864 (31.0%) 828 (6.6%) 696 (5.6%) 496 (4.0%) 1,006 (8.1%) 158 (1.3%)

* *%, percentage of the total group of 12,470 patients*.

With respect to the item scores on the SPI scale (Table 2), patients referred for IHT had lower scores for problems on all items of the SPI scale than patients referred for voluntary admission. For example, the number of patients referred for IHT who were dangerous to others was lower than the number of those referred for voluntary admission. The frequencies of substance use and social complications were lower in the IHT group than in the voluntary admission group. Also smaller proportions of patients referred for IHT had problems with motivation for treatment (2.0 vs. 6.1%), problems with family involvement (2.0 vs 8.4%) and problems with their living conditions (2.7 vs 11.1%).

**Table 2:** SPI of patients assessed by the psychiatric emergency services.

**Severity of psychiatric illness**	**IHT**	**Voluntary admission**	**Total**
Total n*	655 (5.3%)	2,875 (23.0%)	1,2470 (100%)
Suicide potential: *n* Danger to others: *n* Severity of psychiatric symptoms: *n* Problems with self-care: *n* Substance abuse: *n* Medical complications: *n* Social complications: *n* Problems with professional functioning: *n* Problems with living conditions: *n* Problems with motivation for treatment: *n* Problems with compliance: *n* Problems with disease awareness: *n* Problems with family involvement: *n* Persistence of problems: *n*	174 (26.6%) 2 (0.3%) 247 (41.8%) 95 (14.5%) 45 (6.9%) 65 (9.9%) 100 (15.3%) 172 (26.3%) 18 (2.7%) 13 (2.0%) 17 (2.6%) 244 (37.3%) 13 (2.0%) 47 (7.2%)	793 (27.6%) 180 (6.3%) 1,289 (44.8%) 555 (19.3%) 444 (15.4%) 348 (12.1%) 715 (24.9%) 865 (30.1%) 318 (11.1%) 176 (6.1%) 217 (7.5%) 1,310 (45.6%) 242 (8.4%) 531 (18.5%)	2,322 (18.6%) 1,685 (13.5%) 4,945 (39.7%) 2,429 (19.5%) 1,843 (14.8%) 1,573 (12.6%) 2,926 (23.5%) 3,465 (27.8%) 1,304 (10.5%) 1,485 (11.9%) 1,278 (10.2%) 6,362 (51.0%) 1,032 (8.3%) 2,176 (17.4%)

* *%, percentage of the total group of 12,470 patients*.

Table 3 shows the process characteristics of the two groups. At the time of referral, most patients referred for IHT had no mental health practitioner (36) unlike the majority of patients referred for voluntary admission (56.0%).

**Table 3:** Process characteristics of patients assessed by the psychiatric emergency services.

**Factors**	**IHT**	**Voluntary admission**	**Total**
Total n*	655 (5.3%)	2,875 (23.0%)	1,2470 (100%)
Mental health treatment at the time of referral: *n*	236 (36.0%)	1,610 (56.0%)	6,440 (51.6%)
Assessment outside office hours: *n*	237 (36.2%)	1,732 (60.2%)	6,657 (53.4%)
Place of assessment Home: *n* Police station: *n* Family doctor: *n* General hospital: *n* Psychiatric hospital: *n* Other: *n*	338 (51.6%) 9 (1.4%) 11 (1.7%) 28 (4.3%) 222 (33.9%) 47 (7.2%)	935 (32.5%) 316 (11.0%) 105 (3.7%) 319 (11.1%) 880 (30.6%) 320 (11.1%)	4,086 (32.8%) 1,674 (13.4%) 249 (2.0%) 1,357 (10.9%) 3,991 (32.0%) 1,113 (8.9%)
Referrer Family doctor: *n* Police: *n* General hospital: *n* Other: *n*	377 (57.6%) 10 (1.5%) 12 (1.8%) 256 (39.1%)	1,025 (35.7%) 324 (11.3%) 192 (6.7%) 1,334 (46.4%)	3,942 (31.6%) 1,813 (14.5%) 726 (5.8%) 5,989 (48.0%)
Reason for referral Depressive symptoms: *n* Psychotic symptoms: *n* Danger to others: *n* Danger to themselves: *n* Other: *n*	84 (12.8%) 177 (27.0%) 27 (4.1%) 314 (47.9%) 53 (8.1%)	151 (5.3%) 736 (25.6%) 235 (8.2%) 1,574 (54.7%) 179 (6.2%)	611 (4.9%) 3,287 (26.4%) 1,460 (11.7%) 6,100 (48.9%) 1,012 (8.1%)

* *%, percentage of the total group of 12,470 patients*.

While most patients in the IHT group (63.8%) had been assessed during office hours, most in the voluntary admission group (60.2%) had been assessed outside office hours. Majorities of patients in both groups (51.6 vs. 32.5%) had been assessed in their domestic environment. While a substantial proportion of patients in the voluntary admission group had been assessed in a police station (11.0%) or general hospital (11.1%), this was not the case with patients in the IHT group (1.4 vs. 4.3%). In both groups, the main referrer was the family doctor. There were few referrals by the police or a general hospital in the IHT group (1.5 and 1.8%, respectively), but more in the voluntary admission group (14.5 and 5.8%). The main reasons for referral in both the IHT and voluntary groups were danger to themselves (47.9 vs. 54.7%) and psychotic symptoms (27.0 vs. 25.6%). Proportionally, danger to others was a more common reason for referral for voluntary admission (11.7%).

### Multiple Regressions Analyses

In multiple logistic regression analysis, four patient characteristics maintained their association with referral for IHT. Female patients were more likely to be referred for IHT (*OR* = 1.47, 95% *CI* = 1.29-1.67), whereas patients with higher age (*OR* = 0.99, 95% *CI* = 0.98-0.99), problems with motivation for treatment (*OR* = 0.58, 95% *CI* = 0.35-0.96) and problems with family involvement (*OR* = 0.37, 95% *CI* = 0.22-0.62) were less likely to be referred for IHT. Three process characteristics maintained their association: patients referred by a family doctor were more likely to be referred for IHT (*OR* = 1.55, 95% *CI* = 1.35-1.77), whereas patients assessed outside office hours (*OR* = 0.53, 95% *CI* = 0.46-0.61) and with mental health treatment at the time of referral (*OR* = 0.63, 95% *CI* = 0.54-0.72) were less likely to be referred for IHT. Model fit indices showed a reasonable fit to the data, but understanding of referral for IHT could evidently be improved (Nagelkerke's pseudo-R square = 14).

## Discussion

Our comparison of the patient and process characteristics associated with referral for IHT vs. voluntary admission found that most patients referred for IHT were female, were generally the most motivated for treatment, and had families that were more involved. However, because the model fit was restricted, these results should be interpreted with caution. As IHT is a voluntary, home-based approach, safe treatment in a patient's domestic environment depends on collaboration and commitment. We also found that another important condition for home treatment is the availability of involved family members. Most referrals for IHT in our region seemed to result from assessments during office hours, in which the family doctor was the most common referrer. During office hours, IHT practitioners were available to discuss referrals for IHT, possibly lowering the threshold for referral. This finding is comparable to a previous study where patients were more likely to be accepted to psychiatric home treatment if they were referred in normal working hours (13). IHT was a relatively novel intervention in our region, as the first teams in Rotterdam started only in 2015, it is possible that healthcare professionals were cautious about referring patients to it. Neither were most patients referred for IHT receiving mental health treatment at the same time. If a patient was currently not in treatment, referral for IHT was more likely than referral for voluntary admission, suggesting that IHT is an alternative when e.g., waiting lists prohibit direct referral to outpatient care.

Stulz et al. (12) also investigated the triage process of indicating IHT, but mostly after facilitated discharge. They found a primary diagnosis of anxiety or stress-related disorder, employment status and the degree of social problems affected the probability of whether or not HT was initiated. This is not in line with our findings, but could be explained by the different population of the studies.

Two earlier studies did investigate factors associated with admission to psychiatric care after IHT already started (10, 11). e.g., in the United Kingdom, it was found that high suicidal ideation at initial assessment for an IHT team was associated with admission after the IHT treatment started (11). This is in line with the clinical profile we identified, in which patients who were referred for admission more frequently had suicidal ideations. In a second British study, Cotton et al. (10) found that patients were more likely to be admitted to a psychiatric hospital after contact with a crisis-resolution team if they had been uncooperative with initial assessment, were at risk of self-neglect, had a history of compulsory admission, had been assessed outside office hours, and had been assessed in hospitality casualty departments. This is partially in line with our findings that patients who were referred for IHT were relatively more motivated for treatment, and had been assessed during office hours.

In Norway, Hasselberg et al. (22) compared two IHT like teams to identify factors associated with the transfer from IHT back to inpatient care. They found that the patients most likely to be admitted were those with psychotic symptoms, suicidal risk, and a prior history of admissions. Again, these findings are consistent with our finding that patients who were referred to IHT were not currently being treated by a mental health practitioner.

Earlier studies gave no indication that family involvement—a feature of the clinical profile of patients referred for IHT—was a factor that might be associated with referral to IHT. In the UK, however, Brooker et al. (23) investigated the admission decisions that followed contact with the emergency psychiatric outreach services. They found that, after contact with the emergency psychiatric outreach services, low family support was associated with a higher number of hospital admissions. Also in the UK, Brimblecombe et al. (11) found that a small yet notable number of admissions involved patients treated by a crisis resolution and health treatment team because the carers of patients treated at home had been “unable to cope” (8.1%). Finally, this is in line with the findings of Mötteli et al. (24), whose study in Switzerland suggested that that the greatest benefit of IHT was derived by patients with acute mental health problems who had a certain level of social support. Family support thus seems to be an important aspect of effective crisis management in patients' own domestic environment.

## Limitations

Our results must be interpreted with caution since our analyses were based on retrospective routine data. Consequently, acquisition of certain specific data (e.g., intervention outcome) was not possible and should be pursued in future research. The group of patients referred for IHT is relatively small and substantially smaller than the group referred for voluntary admission. The number referred for IHT has increased over time, probably because IHT is still a relatively novel intervention in the Netherlands, and is still finding acceptance with mental health care professionals as a common or standard procedure. As such a process may have influenced our results, a longer period of data collection is needed.

Another limitation of this study is that we only tested the associations between IHT and voluntary admissions, which is what IHT is primarily intended to reduce. Although it would be relevant to establish whether IHT might also reduce the number of involuntary admissions, the observational data we obtained in this study were not suitable for comparing referral to IHT vs. involuntary admission. As the model fit was restricted, not all variance can be explained by the variables measured between the referred patients, which may be due to the relatively novel intervention. While the clinical profile generated in this study provides a first insight into the relevant patient and process characteristics linked to referral, it now needs to be refined. Finally, we should state that this study was carried out in the Rotterdam area, where IHT and the emergency psychiatric outreach services operate as separate departments. The results of this study reflect the referral pattern of the outreach services in this particular region. Referral patterns in other regions may yield different results. In addition, IHT and outreach services in the Rotterdam region were two different and separated services, in contrast to other regions of the Netherlands, where these two services are integrated. Such a separation, which may have biased our results, and also may have affected the generalizability of our findings. For this reason, our clinical profile of IHT patients should only be applied to patients in other IHT teams with the greatest caution.

## Conclusions

The earlier literature supports our findings that, relative to patients referred for voluntary treatment, patients referred for IHT were more motivated for treatment, had their family's support, had been assessed during office hours, had, in most cases, been referred by the family doctor, and were not receiving mental health treatment at the time of referral. Overall, patients referred for IHT had fewer problems before referral than those referred for voluntary admission. IHT also seems to function as a first mental health treatment for patients who do not have a mental health practitioner. It would be relevant to further establish the clinical profile of patients referred for IHT we found, especially the role of the family members involved.

## Data Availability Statement

The raw data supporting the conclusions of this article will be made available by the authors, without undue reservation.

## Author Contributions

GA conceived the study and wrote the manuscript. AW, RW, and CM provided advice on the system design of the database, reviewed, and commented on the manuscript. All authors contributed to the preparation of the article and approved the final version.

## Conflict of Interest

The authors declare that the research was conducted in the absence of any commercial or financial relationships that could be construed as a potential conflict of interest.

## Publisher's Note

All claims expressed in this article are solely those of the authors and do not necessarily represent those of their affiliated organizations, or those of the publisher, the editors and the reviewers. Any product that may be evaluated in this article, or claim that may be made by its manufacturer, is not guaranteed or endorsed by the publisher.
